# Right ventricular outflow tract stenting promotes pulmonary artery development in tetralogy of fallot

**DOI:** 10.3389/fsurg.2023.1056772

**Published:** 2023-02-13

**Authors:** Hui Guo, Zhongshi Wu, Tianli Zhao, Jinfu Yang, Shijun Hu, Can Huang, Yifeng Yang, Li Xie

**Affiliations:** The Department of Cardiovascular Surgery, The Second Xiangya Hospital of Central South University, Central South University, Changsha, China

**Keywords:** tetralogy of fallot, right ventricular outflow tract stent, pulmonary artery, oxygen saturation, primary palliation

## Abstract

**Background:**

Right ventricular outflow tract (RVOT) stenting seems to be suggested as a promising treatment option and an alternative to modified Blalock-Taussig shunt (mBTS) in the initial palliation of patients with Fallot-type lesions in recent years. This study sought to assess the effect of RVOT stenting on the growth of the pulmonary artery (PA) in patients with Tetralogy of Fallot (TOF).

**Methods:**

Retrospective review analyzing 5 patients with Fallot-type congenital heart disease with small pulmonary arteries who underwent palliative with RVOT stenting and 9 patients underwent modified Blalock-Taussig shunt within 9 years period. Differential left PA (LPA) and right PA (RPA) growth was measured by Cardiovascular Computed Tomography Angiography (CTA).

**Results:**

RVOT stenting improved arterial oxygen saturation from median of 60% (interquartile range [IQR]: 37% to 79%) to 95% (87.5% to 97.5%) (*p* = 0.028). The LPA diameter *Z*-score improved from −2.843 (−3.51–2.037) to −0.78 (−2.3305–0.19) (*p* = 0.03), the RPA diameter *Z*-score improved from median −2.843 (−3.51–2.037) to −0.477 (−1.1145–0.459) (*p* = 0.002), the Mc Goon ratio increased from median 1 (0.8–1.105) to 1.32 (1.25–1.98) (*p* = 0.017). There were no procedural complications and all 5 patients have undergone final repair in the RVOT stent group. In the mBTS group, the LPA diameter *Z*-score improved from −1.494 (−2.242–0.6135) to −0.396 (−1.488–1.228) (*p* = 0.15), the RPA diameter *Z*-score improved from median −1.328 (−2.036–0.838) to 0.088 (−0.486–1.223) (*p* = 0.007), and there were 5 patients occur different complications and 4 patients was not attained the standards of final surgical repair.

**Conclusion:**

RVOT stenting, compared with mBTS, seems to better promote pulmonary artery growth, improve arterial oxygen saturations, and have less procedure complications in patients with TOF who being absolute contraindicated for primary repair due to high risks.

## Introduction

Tetralogy of Fallot (TOF) is the most common defect of children with cyanotic heart disease, with a prevalence of approximately 10 percent of congenital heart disease (1). Over the past six decades, outcomes have continued to improve that more than 95% of the newborns with this problem would be expected to survive into adulthood after repair surgery (2, 3). For some patients with tetralogy of Fallot who have appropriate size of pulmonary artery, acceptable cardiac function, and no extracardiac complications, can be candidates for primary surgical repair within 3–9 months after birth, and the postoperative outcome was excellent in these infants. Current improvements in cardiac surgery reduced the patient mortality rate to 0.5%–2.5% throughout time (2–4). However, surgical repair of certain TOF types is challenging. Low body weight, small pulmonary arteries and complex anatomies all are among the risk factors for a terrible outcome, hence primary repair was absolute avoided for such TOF patient (5–7).

Two-stage surgical therapy with growth of pulmonary artery was suggested as an appropriate option (8–11). The indicators for two-stage repair and primary palliation were active cyanosis, low body weight and pulmonary arteries of small size (Mc Goon ratio <1.2 (4). The purpose of primary palliation is to promote the development of pulmonary arteries, increase pulmonary blood flow and improve arterial oxygen saturations, so as to attain the standard of final surgical repair. The preferred palliative surgery in the past is modified Blalock-Taussig shunt (mBTS). However, there are several postoperative complications and a high rate of morbidity and mortality following mBTS (12–14). In recent, right ventricular outflow tract (RVOT) stenting has emerged as an alternative to surgical method of a modified Blalock-Taussig shunt to promoting pulmonary artery growth in cyanotic children with tetralogy of Fallot accompanied with pulmonary arterial dysplasia. In this retrospective research, we sought to assess the effect of RVOT stenting on the growth of the pulmonary arteries in Tetralogy of Fallot patients with high risk factors.

## Methods

This is a 9-year (2012–2021) single-center retrospective review study, including all 14 patients underwent palliation by RVOT stenting or mBTS for TOF-type lesions at the Department of Cardiovascular Surgery at the Second Xiangya Hospital of Central South University. Patient demographics including age, height, weight, non-cardiac comorbidities, stent or shunt diameter, pulmonary artery diameter under cardiovascular CTA, length of ICU stay after palliation, palliation complications, palliate time until final surgical repair, CPB time and X-clamp time at repair in Tables 1, 2. Table 3 includes blood oxygen saturation, PA diameter and *Z*-score, Mc Goon ratio. Pulmonary vessel growth assessed using *Z*-scores derived from the data of Lopez et al. (15). Patient data was collected for assess the risks of right ventricular outflow tract stenting and modified Blalock-Taussig shunt, the impacts on pulmonary artery development in children and the results of final surgery repair. The contrasted time points were pre-palliation, 24 h post palliative operation, and pre-final surgical repair.

**Table 1:** RVOT stenting patient features at palliation and repair surgery.

Patient number	1	2	3	4	5	Total (median, IQR)
Cardiac anatomy	TOF	TOF	TOF	TOF	TOF	/
Non-cardiac comorbidities	/	Prematurity Brain abnormality Cerebral hemorrhage Pulmonary infection	/	/	Brain abnormalities Pulmonary infection Anemia	/
Sex	Male	Male	Male	Male	Male	/
Age at palliation (day)	161	40	72	42	92	72 (41–126)
Age at repair (day)	238	327	234	154	166	234 (160–282.5)
palliation time (day)	77	287	162	112	74	112 (75.5–224.5)
Weight at palliation (kg)	5	2.1	6.8	4.3	3	4.3 (2.55–5.9)
Weight at repair (kg)	6	7	8.7	9	4.5	7 (5.25–8.85)
Stent diameter (mm)	4.5	4.5	4	4	4	4 (4–4.5)
ICU LOS (hour)	40	110	21	23	140	40 (22–125)
CPB time at repair (min)	104	156	177	122	85	122 (94.5–166.5)
X-clamp time at repair in min	67	72	128	85	55	72 (61–106.5)

RVOT, right ventricular outflow tract; TOF, tetralogy of Fallot; IQR, interquartile range; HLHS, hypoplastic left heart syndrome; LOS, length of stay; ICU, intensive care unit; CPB, cardiopulmonary bypass.

**Table 2:** mBTS patient features at palliation and repair surgery.

Patient number	6	7	8	9	10	11	12	13	14	Total (median, IQR)
Cardiac anatomy	TOF	TOF	TOF	TOF	TOF	TOF	TOF	TOF	TOF	/
Sex	Female	Female	Female	Female	Female	Male	Male	Male	Male	/
Age at palliation (day)	395	427	1273	319	732	521	132	382	94	395 (225.5–626.5)
Age at repair (day)	/	750	/	540	1307	940	/	/	248	750 (394–1123.5)
Palliation time (day)	/	323	/	221	757	419	/	/	158	323 (189.5–588)
Weight at palliation (kg)	8	6.5	11	8	9	7.5	6.5	9	6	8 (6.5–9)
Weight at repair (kg)	/	10	/	9.5	13	9	/	/	7.9	9.5 (8.45–11.5)
Shunt diameter (mm)	5	6	6	5	6	5	5	6	5	5 (5–6)
ICU LOS (hour)	102	50	82	75	168	94	/	67	235	88 (69–151.5)
CPB time at repair (min)	/	98	/	87	130	112	/	/	102	102 (92.5–121)
X-clamp time at repair (mm)	/	62	/	56	58	74	/	/	70	62 (57–72)
Palliation complications	Anastomotic stenosis Lung infections Cyanotic spells	/	Anastomotic stenosis Undeveloped LPA	/	Shunt duct thrombosis	/	Ventricular fibrillation Death	Undeveloped PA Shunt duct blocked	/	/
Surgical repair	No	Yes	No	Yes	Yes	Yes	No	No	Yes	/

mBTS, modified Blalock-Taussig shunt; TOF, tetralogy of Fallot; IQR, interquartile range; LOS, length of stay; ICU, intensive care unit; CPB, cardiopulmonary bypass; LPA, left pulmonary artery.

**Table 3:** Clinical and cardiovascular CTA features of the patients before and after palliation and before final surgical repair of TOF.

Features	Before palliation, medians and IQR	After palliation, medians and IQR	Before final surgical repair of TOF, medians and IQR	*P* value
**ROVT stenting**				
Blood oxygen saturation, %	60 (37–79)	95 (87.5–97.5)	83 (73–86.5)	*P*1,2 = 0.008 *P*1,2,3 = 0.005
LPA diameter, mm	3 (2–4.2)		6 (5.55–6.85)	*P*1,3 = 0.001
LPA *Z*-score	−2.843 (−3.51–2.037)		−0.477 (−1.1145–0.459)	*P*1,3 = 0.002
RPA diameter, mm	2.5 (2.1–3.5)		4.5 (4–6.55)	*P*1,3 = 0.021
RPA *Z*-score	−2.892 (−3.54–2.2285)		−0.78 (−2.3305–0.19)	*P*1,3 = 0.03
Mc Goon ratio	1 (0.8–1.105)		1.32 (1.25–1.98)	P1,3 = 0.017
**mBTS**				
Blood oxygen saturation, %	70 (37.5–91)	90 (70–92.5)	93 (73–97)	*P*1,2 = 0.101 *P*1,2,3 = 0.085
LPA diameter, mm	5 (4.25–6.25)		7 (5.75–9)	*P*1,3 = 0.064
LPA *Z*-score	−1.494 (−2.242–0.6135)		−0.396 (−1.488–1.228)	*P*1,3 = 0.15
RPA diameter, mm	5 (4.25–6)		7.3 (6.85–9)	*P*1,3 = 0.005
RPA *Z*-score	−1.328 (−2.036–0.838)		0.088 (−0.486–1.223)	*P*1,3 = 0.007
Mc Goon ratio	1.3 (1.05–1.4)		1.8 (1.75–1.95)	*P*1,3 < 0.0001

LPA, left pulmonary artery; RPA, right pulmonary artery; RVOT, right ventricular outflow tract; mBTS, modified Blalock-Taussig shunt; IQR, interquartile range.

The decision for initial palliation was based on clinical presentation and signs and the development of pulmonary arteries. Our institution prefers primary repair for patients who have appropriate size of pulmonary arteries, acceptable cardiac function and no extracardiac complications. However, palliation will be the first choice for patients with below unfavorable clinical or anatomical conditions, low body weight, active cyanosis, and pulmonary arteries of small size (Mc Goon ratio <1.2), they are the primary indication for palliative intervention in this study. The purpose of the intervention was to promote the growth in diminutive pulmonary arteries to appropriate PA size prior to complete repair, improve cyanosis symptoms and prolong the survival time of infants. In this study, four patients each received expandable bare metal coronary stents of different diameters, and patient four received a drug-eluting stent, nine patients underwent mBTS with appropriate size of shunt tube.

The process of ROVT stenting procedure is that we use interventional guidewire through the right femoral vein, inferior vena cava, right atrium, right ventricle, pulmonary artery route, then select the appropriate stent release position under ultrasound or Digital subtraction angiography (DSA). In patient 2–4, we release the stent under ultrasound, and the others under DSA. The end of the stent is placed at the main pulmonary artery when both pulmonary arteries are dysplasia. When there is only unilateral dysplasia, it is placed at the opening. mBTS surgery is a routine thoracotomy procedure in which an artificial tube is connected from the right subclavian artery to the right pulmonary artery.

### Statistics

Continuous variables were analyzed as median and IQR. Differences in continuous variables between three matched groups (before palliation, after palliation, and before final surgical repair) in ROVTs and mBTS group were tested using One-Way Repeated Measures ANOVA. (IBM SPSS Statistics version 25). These data were tested for normality and conformed to a normal distribution. *P* ≤ 0.05 were regarded as statistically significant.

## Result

### Patient cohort

Five patients (all male) with TOF underwent RVOT stent implantation and nine patients (four male) underwent mBTS over a period of 9 years. Basic patient demographics about palliative intervention and final repair surgery showed in Tables 1, 2. In RVOT stenting group, two patients had brain abnormalities and pulmonary infection, one patient had anemia and another one has been diagnosed with cerebral hemorrhage. In mBTS group, two patients had anastomotic stenosis, two patients with poor pulmonary arteries development, one patient had shunt duct thrombosis and then not founded after anticoagulant therapy, one patient had shunt duct blocked, the angiography revealed no blood flow through to the right pulmonary artery during follow-up, and one patient dead due to ventricular fibrillation during ICU time. Table 3 demonstrates the pulmonary arteries diameter data obtained from cardiovascular CTA and the blood oxygen saturation at palliation period and pre-final repair. All 5 patients underwent transpulmonary valve ROVT stent, with the distal end of the stent placed in the right pulmonary artery or pulmonary artery trunk. Nine patients underwent the surgery that connect the right subclavian artery and the right pulmonary artery by shunt tube. Noteworthy, all patients demonstrated a rapidly increase in blood oxygen saturation after RVOT stent implantation compared with preoperative. Oxygen saturations increased from median of 60% (interquartile range [IQR]: 37% to 79%) pre-operative to 95% (87.5% to 97.5%) post-operative (*p* = 0.028), and at the time of final repair, the patient's blood oxygen saturation still maintained at median 83% (IQR: 73%–86.5%) (*p* = 0.005). The decrease in blood oxygen saturation during palliative periods may be due to in-stent obstruction or the increased hypertrophy of right ventricular outflow tract, or it is related to the relative hypoxia due to the increase in the infant's oxygen demands with the development of the body. In the mBTS group, Oxygen saturations increased from median of 70% (interquartile range [IQR]: 37.5% to 91%) pre-operative to 90% (70% to 92.5%) post-operative (*p* = 0.101), and at the time of final repair, the patient's blood oxygen saturation maintained at median 93% (IQR: 73%–97%) (*p* = 0.085). The increase in oxygen saturation in patients underwent mBTS was no statistically significant (*P *> 0.05). It may be associated with shunt anastomotic stenosis, intra-tube thrombosis, or the increased oxygenated blood flows mainly to the right pulmonary artery and very little to the left pulmonary artery (Table 3). Four patients received expandable bare metal coronary stents in different diameters, and patient 4 received drug-eluting stents. These stents have a median diameter of 4 mm (4–4.5) mm. Nine patients underwent mBTS with a median diameter of 5 mm (5–6) mm shunt tube. All patients were admitted to the ICU for observation and treatment after palliation surgery. There were no surgery-related complications discovered during palliative operation and ICU treatment in palliative ROVT stenting group, one patient in mBTS group died due to ventricular fibrillation.

### PA growth

**Figure 1:**
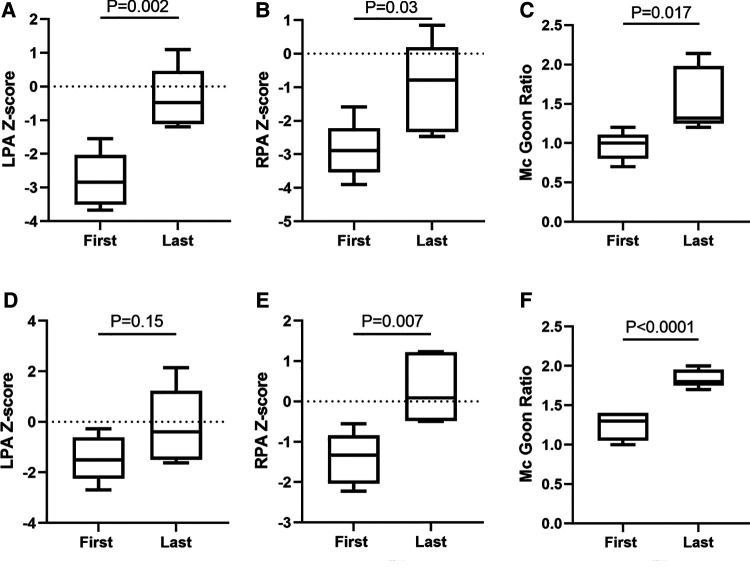
Pulmonary artery growth. Box is first to third quartile. Median is line in box. First, Cardiovascular CTA measure diameter before palliation procedure. Last, Cardiovascular CTA measure diameter before final surgical repair. ROVTs group showed in (**A**-**C**); mBTS group showed in (**D**-**F**).

In ROVTs group, the left pulmonary artery (LPA) diameter increased from median 3 mm (IQR: 2–4.2) mm pre-procedure to median 6 mm (5.55–6.85) mm at the time of final repair (*p* = 0.001), the LPA diameter *Z*-score improved from −2.843 (−3.51–2.037) to −0.78 (−2.3305–0.19) (*p* = 0.03), The right pulmonary artery (RPA) diameter increased from median 2.5 mm (2.1–3.5) mm pre-procedure to median 4.5 mm (4–6.55) mm at the time of surgery (*p* = 0.021), the RPA diameter *Z*-score improved from median −2.843 (−3.51–2.037) to −0.477 (−1.1145–0.459) (*p* = 0.002). The Mc Goon ratio increased from median 1 (0.8–1.105) to 1.32 (1.25–1.98) (*p* = 0.017). In the mBTS group, the LPA diameter increased from median 5 mm (4.25–6.25) mm to 7 mm (5.75–9) mm (*p* = 0.064), the LPA diameter *Z*-score improved from −1.494 (−2.242–0.6135) to −0.396 (−1.488–1.228) (*p* = 0.15). The RPA diameter increased from median 5 mm (4.25–6) mm to 7.3 mm (6.85–9) mm (*p* = 0.005), the RPA diameter *Z*-score improved from median −1.328 (−2.036–0.838) to 0.088 (−0.486–1.223) (*p* = 0.007) (Table 3). Box Plot Diagrams illustrating LPA and RPA *z*-Scores, as well as the Mc Goon ratio, at time of initial palliation (First) and time of surgical repair (Last) in Figure 1.

### Repair

**Figure 2:**
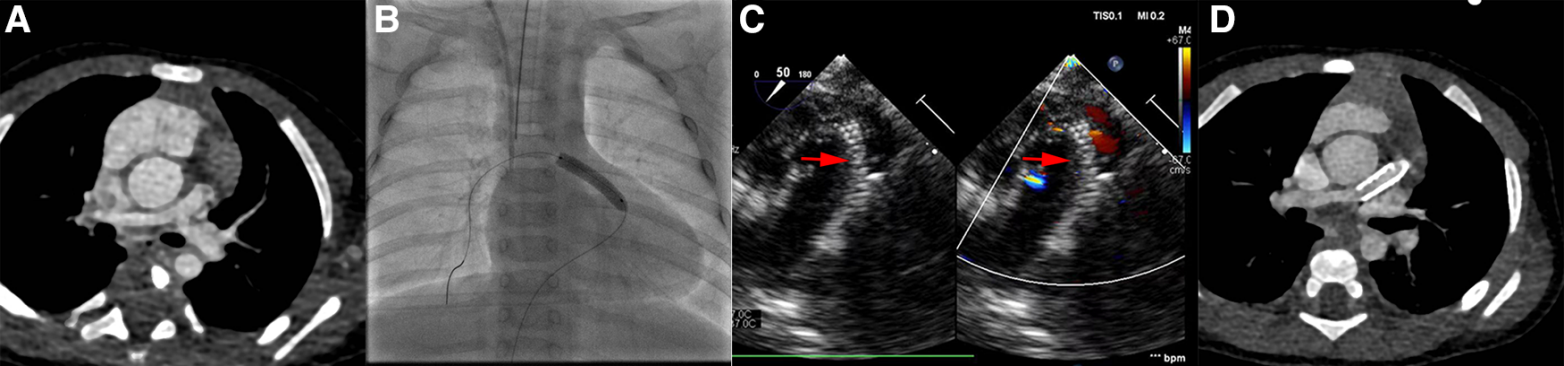
Images in ROVT stenting group. (**A**), patients’ pulmonary artery CTA image before procedure. (**B** and **C**), the pulmonary artery and ROVT stent image during procedure under Digital subtraction angiography or Ultrasound. (**D**), CTA image before final surgical repair. The red arrow indicates the stent shadow.

**Figure 3:**
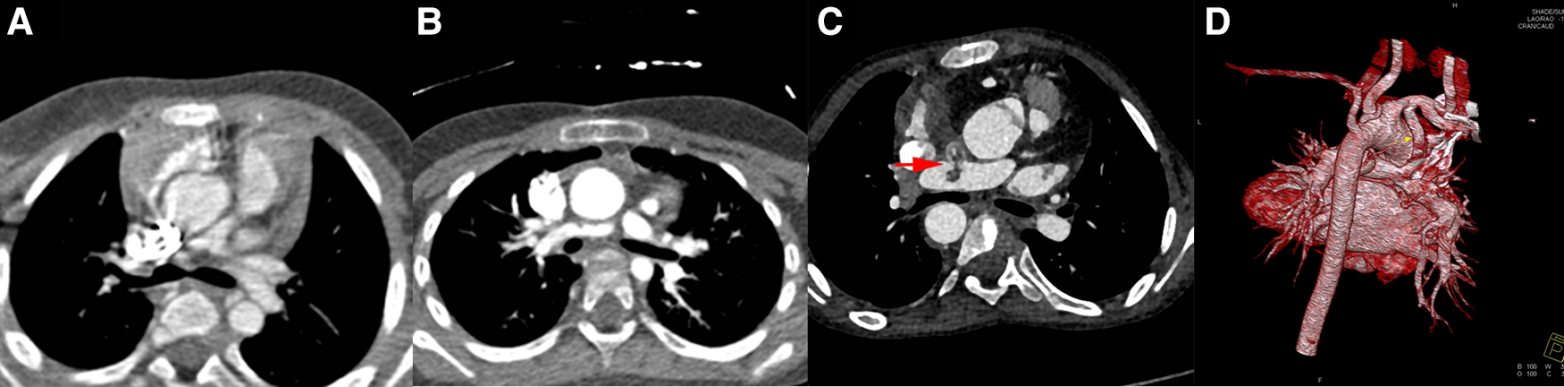
Images in mBTS group. Image (**A**), patients’ pulmonary artery CTA image before procedure; Image (**B**), anastomotic stenosis; Image (**C**), shunt duct and RPA thrombosis; Image (**D**), 3D image before final surgical repair. The red arrow indicates the thrombus.

Within the study period, complete stent or shunt tube was removed from the pulmonary artery without any considerable difficulties in all patients when they attained the final surgical repair standards after palliation procedure. All RVOTs patients underwent pulmonary valve transvalvular widening at the time of surgical repair due to the stent was consistently placed across the hypoplastic pulmonary annulus. In mBTS group, four patients do not attain final surgical standard due to different complications, such as shunt anastomotic stenosis, shunt duct blocked, poor development pulmonary artery and malignant arrhythmia leading to death. One patient had intra-tube thrombosis, the thrombus disappeared after anticoagulant therapy and repair surgery was performed then. The hypertrophic right ventricular outflow tract muscle was removed or the narrowed pulmonary valve was cut open at the time of final surgical repair. Typical Cardiovascular CTA or ultrasound images of the patients before and after palliation procedure are shown in Figures 2, 3.

The median time from initial RVOT stenting to surgery was 112 days (75.5–224.5) days, and median 323 days (189.5–588) day in mBTS group. The median age at surgery was 234 days (160–282.5) days and the median weight was 7 kg (5.25–8.85) kg, median cardiac-bypass time and cross clamp time at final repair were 122 (94.5–166.5) min and 72 (61–106.5) min respectively in ROVTs group (Table 1). In mBTS group, the median age at surgery was 750 days (394–1123.5) days and the median weight was 9.5 kg (8.45–11.5) kg, median cardiac-bypass time and cross clamp time at final repair were 102 min (92.5–121) min and 62 min (57–72) min respectively (Table 2).

## Discussion

This is the first retrospective study of palliative ROVT stenting in infants with Fallot-type congenital heart disease in China. This retrospective review is about cyanotic Tetralogy of Fallot patient with small pulmonary arteries who underwent palliative procedure within nine years from the Department of Cardiovascular Surgery at the Second Xiangya Hospital of Central South University. These patients were initially selected for palliative procedure due to they had pulmonary arteries dysplasia (Mc Goon ratio <1.2), and the difficulty and the high risk of primary repair. An overview of the RVOT stenting strategy used at Birmingham Children's Hospital was previously published (14).

Numerous cases of RVOT stenting in symptomatic patients with Tetralogy of Fallot have been reported (10, 16–19). These reports demonstrate favorable procedural outcomes with low complication rates, and definitive surgical repair have not been impacted by the short-term RVOT stenting. As a result, in many centers, RVOT stenting has become the preferred procedure for symptomatic patients with TOF (20). In this study, all children were discharged from the hospital after stent placement in the right ventricular outflow tract, and no deaths, cardiac or non-cardiac-related complications occurred during hospitalization. Nine patients underwent mBTS and four patients do not attain final surgical standard due to surgical-related complications, such as shunt anastomotic stenosis, shunt duct blocked, poor development pulmonary artery and malignant arrhythmia leading to death. One patient had intra-tube thrombosis, the thrombus disappeared after anticoagulant therapy and repair surgery was performed then. There was an increase in oxygen saturation and LPA diameter and diameter *Z*-score in the RVOTs group, but no statistically significant increase in the mBTS group. This may be due to the fact that the shunt duct connects the right subclavian artery and the right pulmonary artery, then the LPA has less blood flow distribution, resulting in poor development. These data shows that RVOT stenting have a low in-hospital mortality rate, a significant surgical effect, and fewer complications. This situation may be because the systemic venous blood is directed to the pulmonary blood circulation after RVOT stenting, and then result in a more effective oxygen uptake, as well as promote pulmonary arterial growth, and without a reduction in perfusion pressure in diastolic aortic and coronary. This consistent with hemodynamics under physiological conditions, rather than being a pathological condition like mBTS, the increased oxygenated blood flows mainly to the right pulmonary artery and little to the left pulmonary artery, it may occur steal phenomenon which can lead to coronary insufficiency, and affect heart function.

Despite the fact that RVOT stenting to be effective and low risk for most patients, there are some complications that might arise, such as pulmonary artery deformation or stent re-stenosis, stent fracture, stent migration, arrhythmias, stent endocarditis, coronary artery compression and death (20–24). In this paper, compare to blood oxygen saturation after RVOT stenting, the decrease in blood oxygen saturation before final surgery may be caused by partial obstruction of stent, or the increased hypertrophy of right ventricular outflow tract, and it may also related to the relative hypoxia due to the increase in the infant's oxygen demands with the development of the body. Because of the small quantity of TOF patients who meet the standards of ROVT stenting, there is no dedicated stent but coronary stents are generally used. Therefore, the size, length, and material of the stent may not be optimal choice, and it is difficult to design the stent individually. However, according to the imaging results of the cardiovascular CTA, there were no stent migration in any of the five patients, and no one else occur any other complications during palliation period.

Tissue reaction and fibrous tissue growth after bare metal stent implantation in the right ventricular outflow tract is a major problem that cannot be ignored in long-term outcome. This problem may result in in-stent stenosis, requiring further surgical interventions, or possibly preventing the stent from being removed during complete repair. Some studies had shown that there was a higher rate of catheter reintervention after RVOT stenting, as additional re-stenting of the RVOT or re-balloon dilation of the pulmonary arteries and the RVOT stent, or require mBTS procedure in the past (14, 21, 25). However, in recent year, there are published research showed that RVOT stent implantation did not compromise surgical repair significantly and was easily removed in final surgery in short-term implantation. In TOF patients, an over-dilatable drug-eluting stent may overcome this problem in whom desire medium to long-term palliation (26). All 5 patients in this study had complete metal stent removed from the pulmonary artery without any difficulties during complete repair, and they did not require catheter reintervention or diversion to mBTS shunt due to significant in-stent restenosis or stent migration. And final repair was also completed successfully in patient 4 who received expandable drug-eluting stent while the palliation time is 112 days.

Reported transannular patch rates are high (36%–100%) when ROVT stent procedure is performed in infancy, and stent implantation eliminates the possibility of pulmonary valve-reserve repair (27, 28). Therefore, stents should better be implanted in patients with narrow right ventricular outflow tract and eventually require transannular patch repair. The pre-operative pulmonary artery *Z*-score of all five patients in the study meet the standards for transvalvular widening treatment, and after palliative treatment of ROVT stenting, the pulmonary valve was damaged due to stent implantation though the pulmonary artery were well developed, thus they were undergone pulmonary valve transvalvular widening at the time of surgical repair.

While primary complete surgical repair remains the optimal treatment option for tetralogy of Fallot, it is not considered to be the preferred method for those cyanotic patients with small pulmonary arteries. The earliest reports of neonatal primary repair were not encouraging, all of the early deaths occurred in patients with small PAs, and the major post-operative complications included hematogenous, endocarditis, pericardial effusion, chylothorax, post-operative ventilation >7 days, and long ICU stay (16, 29, 30). On the contrary, there are low post-operative risk, fewer complications, and a shorter length of stay in the ICU after ROVT stenting in TOF patients with small PAs. RVOT stenting shows a low post-procedural morbidity and mortality rate compared with neonatal primary repair. It also has a high clinical efficiency in the aspects of achieving proper pulmonary arterial growth during palliation along with uptakes proper arterial O2 saturation, so as to conform to the criterions of final repair sooner. From an economic and social point of view, compared to primary repair, the patients who underwent RVOT stent placement is suitable for infants with low body weight, low oxygen saturation, and poor pulmonary arterial development, because the cost of medical treatment can be reduced, medical resources can be better saved, and the burden on the families of patients and society can be lightened.

Modified Blalock-Taussig shunt (mBTS), the most commonly used palliative surgery for patients with Tetralogy of Fallot in the past, has a high mortality rate of about 7%–8%, and the mortality rate has not decreased significantly for a long term with the development of medicine, which is unusual in the field of cardiac surgery. This may reflect the inherent instability of the shunt-dependent circulation (12, 31). Unlike the situation that systemic venous blood is directed to the pulmonary blood circulation which is consistent with hemodynamics under physiological conditions after ROVT stenting, mBTS is in a pathological state essentially, and it may occur steal phenomenon which can lead to coronary insufficiency. The small size of pipeline anastomotic stoma would form fibrous scars easily, and it also may be twisted or be narrowed especially in infants. As the result, it blocks the artificial pipeline and affects the development of the right pulmonary artery. Complications of mBTS palliation were pulmonary artery tortuosity, chylothorax, phrenic and vocal cord nerve damage, shunt stenosis, hyper-circulation, and death (14, 25, 32). Compared to the follow-up data of the mBTS patients in this article, RVOT stenting has lower surgical risks, mortality and complications rate. The pulmonary artery had development better, and the blood oxygen saturation was significantly improved. Therefore, right ventricular outflow tract stenting instead of Modified Blalock-Taussig shunt appears to be regarded as a safer and more effective method to increase pulmonary blood flow and promote pulmonary artery growth in Tetralogy of Fallot patients with high risks.

The main limitation of this study is that this is a single centre retrospective review and the sample size of the study is small, therefore the results may not be generalized.

## Conclusion

Compared with mBTS, ROVT stenting can promote pulmonary artery growth and improve oxygen saturation better before surgical repair. It seems to be a safe and effective palliative procedure in TOF patients who has high risks and absolutely avoid primary repair. However, these are preliminary results based on small case studies, and to further support this conclusion, larger or multi-center studies are required to obtain more confirmative evidence.

## Data Availability

The raw data supporting the conclusions of this article will be made available by the authors, without undue reservation.
